# Illness cognition and associated socio-demographic and clinical factors in parents of children with leukemia

**DOI:** 10.1186/s40359-024-01798-3

**Published:** 2024-05-23

**Authors:** Jing Han, Li Zhang, Feng Yang, Linlin Wang

**Affiliations:** 1 School of Nursing, Xuzhou Medical University, Xuzhou, 221004 China; 2 Affiliated Hospital of Xuzhou Medical University, Xuzhou, China

**Keywords:** Leukemia, Pediatric oncology, Parent, Illness cognition, Nursing

## Abstract

**Purpose:**

Illness cognition is an important mediator between psychological and behavioral adjustment and the quality of life for patients and their caregivers. Evidence related to illness cognition among parents of children with leukemia is limited. The purpose of this study is to explore the illness cognition status and associated factors in parents of children with leukemia.

**Methods:**

A cross-sectional survey was conducted with the parents of 335 children with leukemia from three general children’s hospitals in China from January to December 2022. A parents’ version of the illness cognition questionnaire was used to collect data. This included three subscales: helplessness, acceptance, and perceived benefits.

**Results:**

The mean scores of helplessness, acceptance and perceived benefits of parents regarding their children’s disease were 15.56 (4.60), 16.25 (4.41), and 19.96 (3.69) respectively. The multiple regression model indicated seven factors associated with the parents’ illness cognition (adjusted R [2] ranged from 0.182 to 0.134): four socio-demographic factors (parent’s age, role, education level, and family income) and three clinical factors (length of time spent each day caring for the child, the child’s age at diagnosis, and the duration of the disease).

**Conclusion:**

This study reports on different levels of illness cognition and associated factors among parents of children with leukemia. The results may help pediatric oncology medical staff identify risk factors for poor psychological adjustment to children’s diseases. Parents may benefit from psychological support aimed at improving positive illness cognition.

## Introduction

Leukemia is the most common type of pediatric malignancy, accounting for one-third of all childhood cancers [1]. Acute lymphoblastic leukemia (ALL) is the most common subtype, accounting for 75% of leukemia cases and 26% of childhood cancer cases in children under the age of 15 [2, 3]. Progress in developing treatments and advances in supportive care has led to the 5-year survival rate rising to 90% [4]. The length of therapy for most leukemia treatments is two to three years, and it comes with serious side effects that continue to impair the quality of life even after treatment is completed [5]. Parents of children with leukemia face high levels of psychological stress and a heavy economic burden [6]. They report psychological problems, such as distress, anxiety, depression, and helplessness [7]. Chen et al. found that 64.5% of parents of children with leukemia had serious anxiety symptoms [8]. Wakefield et al. found that even 1 year after a child completed treatment, parents were still worried about a relapse or the death of their children [9].

**Fig. 1:**
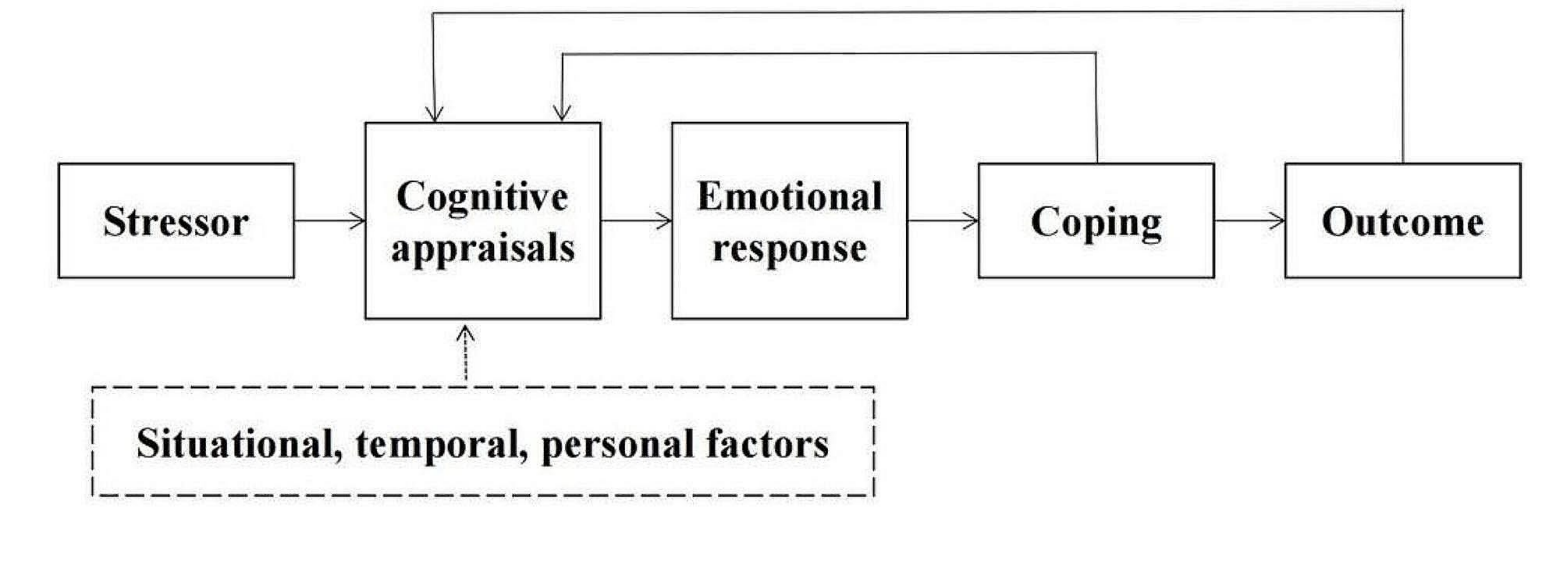
Lazarus’s Transactional Model (Lazarus & Folkman, 1987)

Lazarus’ transactional model claims that stress processes are transactional and coping outcome is informed by both cognitive appraisal of the stressor and the individual’s emotional response [10]. Cognitive appraisals are influenced by situational, temporal, and personal factors [11]. Figure 1 shows the model. Cognitive appraisals are made when confronted with a stressful situation, such as cancer. A cancer diagnosis in children is a stressor for parents, and their response and coping strategy mainly depends on their cognitive appraisal of their children’s disease. There is much evidence demonstrating the associations between coping and outcome (such as psychological distress, depression, quality of life, etc. ) in cancer patients and their caregivers [12–14]. But it is suggested that the cognition appraisal may offer the most predictive value on outcome [15].

Illness cognition refers to a series of psychological responses, such as acceptance, helplesness, perceived benefits, when faced with disease, based on an individual’s appraisal of the disease and state of health [16]. Illness cognition is a significant mediator between disease and well-being [17]. The parents’ cognition of their child’s disease is related to their psychological flexibility and coping strategies, which, in turn, predicts how well the family will adjust over time [18, 19]. Parents are usually the main source of information on their child’s physical and psychological state for medical staff [20, 21]. Previous studies have found that parents’ illness cognition affects their response to their child’s treatment, which directly impacts the child’s quality of life and prognosis [16, 22].

Illness cognition is associated with patients’ socio-demographic variables (such as gender and age) and disease characteristics (such as disease severity and duration). Young male adults with chronic illness perceived fewer benefits and more helplessness than female counterparts [23]. Compared with younger patients with breast cancer, older patients reported a higher level of illness acceptance [24]. Verhoof et al. found that among young adults with a chronic disease since childhood, those who had worse self-care ability perceived less acceptance and more helplessness than those with better self-care ability [23]. For breast cancer patients, illness cognition has been found to become more positive over time [24]. Several questionnaires assess illness cognition, most of which focused on negative illness cognition [25]. The parent’s version of the Illness Cognition Questionnaire (ICQ-P) is a multidimensional measurement of appraising illness representations that involves assessing both positive and negative illness cognition, namely, helplessness, acceptance, and perceived benefits [26].

Although it is widely accepted that illness cognition is an important factor in the well-being of patients and their families, illness cognition has not previously been investigated among parents of children with leukemia. In addition, it is not clear which socio-demographic and disease characteristics are associated with parents’ illness cognition. Understanding illness cognition in parents of children with leukemia have beneficial effects on clinical nursing practice, such as the promotion of parents’ psychological adjustment and active cooperation with treatment. This study aimed to evaluate the illness cognition of parents of children with leukemia, as well as the associated socio-demographic and clinical factors. It is anticipated that findings will help identify parents who might get the most benefit from nursing support directed at problematic cognition.

## Methods

### Design

A cross-sectional survey design was used and data were collected between January and December 2022.

### Participants

A convenience sampling method was used to recruit parents in three children’s hospitals in eastern China. The inclusion criteria were: having a child under 18 diagnosed with leukemia, having awareness of the child’s disease, having a smartphone, having no cognitive impairments, and having the ability to express opinions. The formula n=(Z_α_^2^×σ^2^)/δ^2^ was used to calculate the sample size to ensure appropriate statistical power, where α = 0.05, Z_α_ = 1.96, σ = 4.45 (pre-experiment results), δ = 0.5. The calculated sample size was 304. We increased the sample size by a further 20% to allow for attrition, so 365 participants were recruited.

### Data collection

Notices about the study were posted in the pediatric oncology units. Any parents who were interested in the study could contact the investigators through WeChat (a social media app in China). After a telephone interview to screen for eligibility, participants signed their consent via their smartphone. Participants were invited to complete a 10-minute-long questionnaire that was uploaded onto the online survey platform Wenjuanxing. During the completion of the questionnaires, a researcher (LZ) was available to answer any questions about the questionnaire. Participants who completed the entire questionnaire received an e-voucher worth $5.

### Instruments

Data collection was carried out using a two-part questionnaire. The first part was a general data questionnaire, including the parent’s socio-demographic factors and the child’s clinical factors. The parent’s data included: role (father/ mother), age, number of children, marital status, education level, work status, household income, and time spent each day caring for the ill child. The child’s data included: gender, age at diagnosis, type of leukemia, disease duration, treatment methods, treatment status, number of symptoms (including anemia, bleeding, infection, and fever), medical insurance, and the family history of cancer.

The second part was the ICQ-P. The ICQ-P was developed by Sint Nicolaas et al. [26], and it is used to evaluate three cognitive aspects of a child’s disease: helplessness, acceptance, and perceived benefits. Helplessness involves focusing on the negative changes associated with the disease and generalizing them to functioning in daily life, e.g. “Because of my child’s illness, I miss the things I like to do most.” Acceptance is associated with acknowledging being ill and perceiving an ability to manage the negative consequences of the disease, e.g. “I can handle the problems related to my child’s illness.” Perceived benefits are related to perceiving positive, long-term consequences of the disease, e.g. “Dealing with my child’s illness has made me a stronger person.” The ICQ-P is an 18-item questionnaire with items rated using a four-point Likert scale (based on the extent to which one agrees with each item, from ‘not at all’ to ‘completely’). Each subscale has six items, which are scored independently, ranging from 6 to 24. The higher the score, the higher the level of the corresponding illness cognition.

After being granted permission by Sint Nicolaas, the questionnaire was translated from English into Chinese and back-translated to guarantee the accuracy in language. First, two Chinese psychiatric doctors proficient in medical and psychological English were invited to translate the original questionnaire independently. Second, the research group compared and integrated the translation drafts and discussed with the two translators to form the initial Chinese translation draft. Third, two native English speakers with a background in psychology and medicine were invited to back-translate the initial Chinese translation draft. Then, the research group compared the two back-translation English drafts and the original English ICQ-P, and discussed with the two back translators to determine the Chinese words. Finally, the research group and the four translators discussed and modified the initial Chinese translation draft, and obtained the Chinese version of ICQ-P. The items and order of the Chinese ICQ-P were consistent with the English ICQ-P. Chinese validation of the ICQ-P was conducted among 394 parents of children with leukemia or other cancer from May 2020 to April 2021. The Cronbach’s alpha for the three subscales ranged from 0.779 to 0.827, and the content validity index was 1.0 and the construct validity was acceptable (Chi-squared/degrees of freedom: 2.306; root mean square error of approximation: 0.077; comparative fit index: 0.902) [27].

### Statistical analysis

Statistical analysis was performed using SPSS (China) version 23.0. All socio-demographic, clinical, and ICQ-P data were analyzed using descriptive statistics. For socio-demographic and clinical data, the number of cases and percentage were used to describe them. The means, and standard deviations (SD) were used to describe the ores of ICQ-P. The Shapiro-Wilk test was used to examine the normality of ICQ-P scores. Scores of ICQ-P (helplessness, acceptance, and perceived benefits) between different groups were performed in univariate analysis, including independent sample t-test, analysis of variance (ANOVA), or Mann–Whitney U test, as appropriate. Variables with *p* < 0.05 in univariate analysis were entered into multiple regression analysis. Multiple stepwise regression analysis was conducted to explore the factors influencing illness cognition. We used dummy variables to represent the categorical variables. *P* < 0.05 was considered statistically significant. Adjusted R [2] was used to describe the regression model, and adjusted R^2^ > 0.7 means high fitting effect, adjusted R [2] ranged from 0.4 to 0.7 means medium fitting effect, and adjusted R^2^ < 0.4 means small fitting effect. G*Power version 3.1 software was used to calculate the power analysis. Based on the effect size with a total sample of 335 patients, the power is 0.95.

## Results

### Characteristics of participants

A total of 365 questionnaires were collected in this study, and 335 parents aged between 20 and 47 completed the questionnaires (a response rate of 91.8%). Sixteen parents withdrew after consent and 14 parents did not complete the questionnaire. Of those who completed the questionnaire, 109 were fathers and 226 were mothers. Nearly 71% of the children covered by the study were diagnosed with ALL and were aged between 1 and 15 years. The socio-demographic and clinical factors of the 335 participants are shown in Table 1.

**Table 1:** Socio-demographic and Clinical Variables (*n* = 335)

Parents’ Variable	*N* (%)	Children’s Variable	*N* (%)
Role		Gender	
Father	109 (32.5)	Boy	188 (56.1)
Mother	226 (67.5)	Girl	147 (43.9)
Age (year)		Age at diagnosis (year)	
20–29	52 (15.5)	< 3	69 (20.6)
30–39	227 (67.8)	3–7	148 (44.2)
40–47	56 (16.7)	8–12	93 (27.8)
No. of children		13–15	25 (7.5)
1	91 (27.2)	Leukemia type	
≥ 2	244 (72.8)	ALL	239 (71.3)
Marital status		AML	57 (17.0)
Married, living together	311 (92.8)	Others	39 (11.6)
Married, separated	10 (3.0)	Disease duration (months)	
Divorced or widowed	14 (4.2)	< 6	157 (46.9)
Education level		6–12	88 (26.3)
Low (≤ 9th grade )	161(48.1)	> 12	90 (26.9)
High (> 9th grade)	174 (51.9)	Treatment methods	
Work status		Chemotherapy	327 (97.6)
Continue working	135 (40.3)	Transplantation	6 (1.8)
Resignation	113 (33.7)	Radiotherapy	2 (0.6)
Asking for leave	87 (26.0)	Treatment status	
Monthly family income per capita (RMB)		Receiving treatment	291 (86.9)
< 1000	104 (31.0)	Completed treatment	44 (13.1)
1000–2999	116 (34.6)	No. of symptoms	
3000–4999	82 (24.5)	1	270 (80.6)
≥ 5000	33 (9.9)	2	52 (15.5)
Daily caring time (hours)		≥ 3	13 (3.9)
< 6	16 (4.8)	Medical insurerance	
6–12	47 (14.0)	Yes	227 (82.7)
12–18	44 (13.1)	No	58 (17.3)
18–24	228 (68.1)	Family history of cancer	
		Yes	17 (5.1)
		No	318 (94.9)

ALL: acute lymphoblastic leukemia; AML: acute myeloid leukemia

### ICQ-P subscale scores of the participants

The range of potential scores for each subscale was 0–24. The scores of three ICQ-P subscales were normality (helplessness: W = 0.993, *p* > 0.05; acceptance: W = 0.917, *p* > 0.05; perceived benefits: W = 0.942, *p* > 0.05). The mean score was 15.56 (SD = 4.60) for helplessness, 16.25 (SD = 4.41) for acceptance, and 19.96 (SD = 3.69) for perceived benefits. The centesimal scores (centesimal score = [mean score/possible highest score]*100) were calculated to compare subscale values, 64.83 for helplessness, 67.71 for acceptance, and 83.17 for perceived benefits.

### Associated socio-demographic and clinical factors of illness cognition

The results of the univariate analysis to identify factors associated with helplessness, acceptance, and perceived benefits are shown in Table 2. There were seven factors associated with helplessness (*p* < 0.05): parent’s role, age, education level, work status, household monthly income, child’s age at diagnosis, and disease duration; three factors associated with acceptance (*p* < 0.05): parent’s role, daily caring time, and child’s age at diagnosis; three factors associated with perceived benefits (*p* < 0.05): daily caring time, child’s age at diagnosis, and disease duration.

The results of multiple regression analysis of factors associated with scores of the three ICQ-P subscales are shown in Table 3. Five variables were entered into the helplessness regression model: parent’s age, education level, household monthly income, child’s age at diagnosis, and disease duration (Adjusted R^2^ = 0.182). The parent’s age had the strongest positive association with helplessness (coefficient = 0.288). The parent’s education level was also positively correlated with helplessness. The monthly family income, child’s age at diagnosis, and child’s disease duration were negatively correlated with helplessness. Three variables were entered into the acceptance regression model: the parent’s role, daily caring time, and the child’s age at diagnosis (Adjusted R^2^ = 0.145). Parent’s daily caring time had the strongest positive association with acceptance (coefficient = 0.342). The parent’s role (mother) and the child’s age at diagnosis were negatively correlated with acceptance. Two variables were entered into the perceived benefits regression model (Adjusted R^2^ = 0.134). The child’s age at diagnosis had the strongest negative association with perceived benefits (coefficient = -0.233), while the disease duration was positively associated with perceived benefits.

**Table 2:** Univariate analysis on the ICQ-P in Parents of Children with Leukemia (*n* = 335)

Variable	Helplessness			Acceptance			Perceived benefits		
	Mean (SD)	t/F/U	*p*	Mean (SD)	t/F/U	*p*	Mean (SD)	t/F/U	*p*
**Parent’**									
Role		2.577	0.010*	0.024*	2.269	0.024*		1.453	0.147
Father	16.49 ± 4.63			17.04 ± 4.75			20.37 ± 3.99		
Mother	15.11 ± 4.53			15.88 ± 4.20			19.75 ± 3.53		
Age (year)		9.626	0.000*		1.269	0.282		0.130	0.878
20–29	13.06 ± 4.36			15.82 ± 4.60			20.12 ± 4.01		
30–39	15.98 ± 4.72			16.15 ± 4.40			19.89 ± 3.55		
40–47	16.18 ± 4.66			17.07 ± 4.27			20.16 ± 3.98		
No. of children		-1.368	0.173		-0.873	0.403		-0.430	0.668
1	15.03 ± 4.14			15.92 ± 4.12			19.81 ± 4.11		
≥ 2	15.76 ± 4.75			16.38 ± 4.51			20.01 ± 3.53		
Marital status		0.815	0.443		0.192	0.825		0.598	0.550
Married, living together	15.55 ± 4.60			16.27 ± 4.42			19.99 ± 3.68		
Married, separated	14.30 ± 4.37			15.40 ± 4.77			18.70 ± 4.27		
Divorced or widowed	16.71 ± 4.92			16.29 ± 4.30			20.07 ± 3.60		
Education level		17.87	0.000*		5.020	0.170		1.024	0.382
Low (≤ 9th grade )	16.42 ± 4.87			17.14 ± 2.95			19.60 ± 3.97		
High (> 9th grade)	13.40 ± 3.46			16.11 ± 4.56			20.11 ± 3.02		
Work status		9.006	0.029*		1.797	0.148		1.051	0.370
Continue working	14.06 ± 4.63			16.34 ± 4.52			20.11 ± 4.78		
Resignation	15.28 ± 3.03			15.85 ± 4.23			19.57 ± 4.12		
Asking for leave	16.27 ± 4.88			15.32 ± 4.02			19.46 ± 3.25		
Monthly family income per capita (RMB)		5.839	0.001*		2.629	0.050		5.972	0.113
< 1000	16.91 ± 4.63			15.59 ± 4.96			19.55 ± 3.89		
1000–2999	15.18 ± 4.49			16.34 ± 4.08			19.55 ± 4.05		
3000–4999	15.21 ± 4.09			16.26 ± 4.15			20.61 ± 3.05		
≥ 5000	13.52 ± 5.11			18.03 ± 3.99			21.03 ± 2.72		
Daily caring time (hours)		0.974	0.405	0.023*	3.226	0.023*	0.004*	4.572	0.004*
< 6	14.50 ± 4.86			14.37 ± 4.17			20.38 ± 4.21		
6–12	15.23 ± 4.18			15.02 ± 4.22			18.30 ± 4.26		
12–18	14.86 ± 4.00			15.84 ± 3.82			20.98 ± 3.25		
18–24	15.83 ± 4.76			16.72 ± 4.51			20.07 ± 3.52		
**Child’s**									
Gender		-0.729	0.466		-0.624	0.533		1.163	0.246
boy	15.40 ± 4.46			16.11 ± 3.97			20.17 ± 3.13		
girl	15.77 ± 4.78			16.43 ± 4.93			19.68 ± 4.29		
Age at diagnosis (year)		6.642	0.000*	0.001*	5.628	0.001*	0.005*	12.976	0.005*
< 3	15.74 ± 4.28			16.55 ± 4.82			20.62 ± 3.27		
3–7	14.90 ± 4.50			15.97 ± 4.04			19.80 ± 3.48		
8–12	17.06 ± 4.48			17.26 ± 4.20			20.63 ± 2.89		
13–15	13.36 ± 5.14			13.40 ± 4.94			16.48 ± 6.08		
Leukemia type		0.916	0.401		0.387	0.824		1.869	0.393
ALL	15.37 ± 4.70			16.39 ± 4.09			20.13 ± 3.38		
AML	15.81 ± 4.73			15.96 ± 5.18			10.95 ± 4.88		
Others	16.38 ± 3.72			15.85 ± 5.17			20.36 ± 3.30		
Disease duration (months)		3.825	0.023*		2.724	0.067	0.017*	8.145	0.017*
< 6	16.67 ± 4.65			15.99 ± 4.39			19.27 ± 4.41		
6–12	15.40 ± 4.01			15.78 ± 4.42			20.07 ± 2.87		
> 12	15.01 ± 4.80			17.17 ± 4.37			21.03 ± 2.64		
Treatment methods		2.824	0.061		0.159	0.853		1.025	0.360
Chemotherapy	15.54 ± 4.60			16.26 ± 4.39			19.91 ± 3.70		
Transplantation	12.16 ± 3.60			16.33 ± 4.27			21.17 ± 3.54		
Radiotherapy	20.50 ± 0.71			14.50 ± 10.61			23.00 ± 1.41		
Treatment status		0.126	0.882		0.814	0.444		1.416	0.244
Receiving treatment	15.61 ± 4.67			16.13 ± 4.40			19.82 ± 3.73		
Completed treatment	15.35 ± 3.76			17.04 ± 4.70			20.70 ± 3.70		
No. of symptoms		2.439	0.089		0.935	0.394		0.480	0.619
1	15.35 ± 4.64			16.27 ± 4.39			19.90 ± 3.57		
2	16.06 ± 4.39			15.83 ± 4.54			20.00 ± 4.24		
≥ 3	18.00 ± 3.92			17.69 ± 4.50			20.92 ± 3.82		
Medical insurance		-1.968	0.050		0.743	0.458		1.191	0.235
Yes	15.54 ± 4.64			16.34 ± 4.46			20.06 ± 3.78		
No	16.64 ± 4.30			15.86 ± 4.19			19.43 ± 3.20		
Family history of cancer		-0.349	0.727		1.430	0.154		-0.928	0.354
Yes	15.94 ± 5.30			16.33 ± 4.36			20.07 ± 3.78		
No	15.54 ± 4.57			14.76 ± 5.23			19.43 ± 3.20		

The total score for each subscale: 24; *: *p*-values for the significant variables

**Table 3:** Multiple regression analysis of the ICQ-P data (*n* = 335)

Dimension	Variables	Coef.	Standardized error	Standardized coef.	t	*p*	Adjusted *R* [2]
Helplessness							18.2%
	Constant	13.331	1.271		10.488	0.000	
	Parent’s age(30–39 years)	2.835	0.657	0.288	4.317	0.000	
	Parent’s age(40–47 years)	2.647	0.845	0.215	3.132	0.002	
	Parent’s education level (≤ 9th grade )	2.077	0.915	0.226	2.270	0.024	
	Monthly family income per capita (RMB 3000–4999 )	-1.776	0.597	-0.184	-2.976	0.00	
	Monthly family income per capita (RMB ≥ 5000 )	-2.429	0.946	-0.158	-2.566	0.011	
	Child’s age at diagnosis(13–18 years)	-3.041	1.030	-0.174	-2.953	0.003	
	Child’s disease duration (> 12 months)	-1.753	0.560	-0.169	-3.132	0.002	
Acceptance							14.5%
	Constant	14.588	1.183		12.331	0.000	
	Parent’s role(mother)	-1.452	0.525	-0.154	-2.764	0.006	
	Parent’s daily caring time(18–24 h)	3.231	1.133	0.342	2.852	0.005	
	Child’s age at diagnosis(13–18 years)	-2.608	1.016	-0.156	-2.567	0.011	
Perceived benefits							13.4%
	Constant	20.020	0.973		20.567	0.000	
	Child’s age at diagnosis(13–18 years)	-3.270	0.831	-0.233	-3.936	0.000	
	Child’s disease duration(> 12 months)	1.870	0.465	0.225	4.018	0.000	

## Discussion

This is the first study that examined illness cognition in parents of children with leukemia. Compared with the negative illness cognition (helplessness), the positive illness cognition (perceived benefits and acceptance) scored higher among parents of children with leukemia. Age, role, education level, household monthly income, daily caring time, and child’s diagnosed age and disease duration were associated with parents’ illness cognition about their children’s leukemia. Although the effect sizes for the results of the analyses are small, this study suggests that clinical staff can use the ICQ-P to identify patients who have negative illness cognition. Meanwhile, this study highlights the importance of evaluating the socio-demographic and clinical contexts of parents and children since this evaluation allows the identification of risk factors for a poorer emotional adjustment to children’s disease.

The score of perceived benefits was the highest among scores of the three subscales in this study. There may be several reasons. Firstly, with the improving treatment and care of leukemia, children endure fewer symptoms during therapy, and this could help to relieve a parent’s psychological distress. The high survival rate and positive prognosis of leukemia could make parents feel more hopeful about their child’s future [2]. Secondly, social support and positive information from family members and healthcare professionals families could strengthen a parent’s positive illness cognition [28, 29]. Thirdly, parents who have experienced significant events and have ‘grown’ from the trauma might reassess the important things in life and find new value in life [30]. This could lead parents to see benefits from the child’s disease.

Compared to those under 30, parents aged over 30 reported higher helplessness. Meanwhile, parents with a lower education level (less than 9th grade) reported high helplessness. This is consistent with previous studies which found that younger parents of children with cancer reported less psychological distress [31]. It was reported that young parents usually put on a happy face and adopted positive coping strategies [32], which could protect parents’ positive cognition and perceived lower helplessness. Wu et al. found that parents of children with leukemia with high education level had a strong caring ability and were more likely to obtain resources such as disease information and caring skills more effectively [33]. Srivastava et al. found that parents with lower education suffered more frequently from severe anxiety [34]. This ability to seek information and support might help parents with a higher education level to feel less helpless.

We found that parents with a higher income had lower helplessness. Compared to those with a monthly family income per capita under 3000 RMB($500), parents with a monthly family income per capita over 3000 RMB reported less helplessness. Santacroce & Kneipp found that 63.6% of parents said pediatric cancer had severely affected their finances, and more than one-half of parents reported that the financial burden severely affected their distress and other stress-related symptoms, such as anxiety, and depression [35]. A higher family income could help parents access more treatment without affecting their household budget, which could alleviate the feeling of helplessness.

In this study, we found that the diagnosed age of children over 13 was negatively associated with their parents’ helplessness, acceptance, and perceived benefits. Compared to those with children diagnosed under 13, parents whose children have been diagnosed with leukemia at over 13 years of age reported lower helplessness, acceptance, and perceived benefits. Nam et al. investigated 366 pediatric cancer caregivers and found that the older the child, the less the caregiver’s burden, which could relieve parental helplessness [36]. Compared with the effect on younger children, cancer treatment can create more problems for older children, such as issues with school life or study, and friendships [37]. Faced with a growing child, parents may feel more worried about that child’s future. Older children have developed a higher cognitive ability, which can also make them realize the dangers of the disease. Adolescents with cancer tend to be negative when facing the disease and treatment side effects, and this can affect their parents’ cognition [38], leading to low acceptance and low perceived benefits around their child’s cancer.

In this study, we found that the disease duration of children over 12 months was negatively associated with their parents’ helplessness, and positively associated with perceived benefits. Parents whose children had the disease for more than 12 months reported lower helplessness and higher perceived benefits compared to those where the illness had lasted less than 12 months. Al-Gamal & Long also found that parents of children with leukemia reported perceived benefits, less anticipatory sadness, and less helplessness with increasing disease duration [39]. Children with cancer and their parents can find bright sides and support for coping, such as different perspectives on life, faith, positive thoughts, and family assistance [40]. The average duration of treatment in leukemia, such as acute lymphoblastic leukemia and acute myeloid leukemia, ranges between 1 and 2.5 years [41]. Most children with leukemia are receiving maintenance therapy after being diagnosed 12 months, and their conditions become stable [42]. Along with the children’s disease duration increased, parents’ post-traumatic growth improved, and psychological distress and catastrophic thoughts decreased [43]. These may alleviate parents’ feelings of helplessness and develop their perceived benefits.

We found that mothers reported lower acceptance than fathers, and parents who spent more than 18 h a day caring for an ill child reported high acceptance of the child’s leukemia. Wikman et al. found that mothers reported more physical and psychological problems than fathers, and their symptoms of depression and posttraumatic stress were associated with more severe symptoms of anxiety [44]. Qualitative research also found that mothers were more vulnerable, needed longer to adapt, were more distressed, and had more negative illness cognition than fathers [45, 46]. Liu et al. found that the longer the child’s hematological disease duration, the lower the level of anticipatory sadness in the caregivers [47]. Through caring for the child, parents learn about the disease and treatment and can master caring skills, they may see the child’s condition more clearly, which may relieve uncertainty and improve their acceptance of the child’s disease.

In this study, we did not find the association between parent’s role and work status and helplessness, and the association between parent’s daily caring time and perceived benefits, although these variables were significant in univariate analysis. There may be two reasons. Firstly, there is an unbalanced gender ratio: female participants are twice as many as males, which might lead to negative statistical results. Secondly, the univariate analysis could help to screen significant variables but often be influenced by mixed factors. The parent’s work status may affect family income, and the parent’s daily caring time might be related to disease duration. These could be adjusted in multiple regression analysis.

### Study limitations

A few factors may have impacted the study results. First, the targeted questionnaire (ICQ-P) is translated from English, and the translation may have had inaccuracy in changing the original questionnaire. The ICQ-P with a majority of positive questions may influence respondents to a more positive overall response. Second, this study was carried out in three general children’s hospitals in eastern China, where the economy and level of medicine are higher than in central and western regions. The results, therefore, may be limited in their generalizability to other locations. Third, a convenience sampling approach was employed in this study, which may have resulted in poor representativeness and heterogeneity of the sample. A randomized sampling method is recommended ensuring the representativeness of the samples and reduce bias. Fourth, this study did not include other factors influencing an individual’s illness cognition. In future research, factors such as physical function, psychological status, and social support should be included to find the important factors affecting parents’ illness cognition. Fifth, this study used a cross-sectional design and cannot identify dynamic illness cognition over time in parents of children with leukemia. A longitudinal study design is recommended for future research.

## Conclusion

This cross-sectional, correlational survey aimed to examine the illness cognition of parents of children with leukemia. We found that, overall, parents reported high perceived benefits of positive illness cognition. Age at diagnosis of more than 13 was negatively associated with helplessness, acceptance, and perceived benefits. Disease duration of more than 12 months was negatively associated with helplessness and positively associated with perceived benefits. The parent’s age of more than 30 and low education level was positively associated with helplessness, while high family income was negatively associated with helplessness. Mothers of children with leukemia reported less acceptance than fathers, and the amount of time spent caring each day was positively associated with acceptance. The results of this study could help pediatric oncology clinical staff identify the risk factors for poor emotional adjustment to leukemia. The assessment of parents’ illness cognition is recommended to be a standard of practice in pediatric oncology units and the ICQ-P is the recommended tool. The findings could also help them identify parents who should be prioritized for screening for emotional distress, and those who may benefit from interventions aimed at improving positive illness cognition. Pediatric oncology professionals play an important role in the provision of psychosocial support. Problem-solving skills training, psychological intervention (such as cognitive behavior therapy), and peer support groups are recommended to help parents of leukemia children cope with the disease and related problems effectively.

## Data Availability

The data of this study are available on reasonable request from the corresponding author.

## Abbreviations

ALL: Acute lymphoblastic leukemiaL
AML: Acute myeloid leukemia
ICQ-P: Parent’s version of the Illness Cognition Questionnaire
SD: Standard deviations

## References

[1] Marcotte EL, Thomopoulos TP, Infante-Rivard C, Clavel J, Petridou ET, Schüz J, Ezzat S, Dockerty JD, Metayer C, Magnani C, Scheurer ME, Mueller BA, Mora AM, Wesseling C, Skalkidou A, Rashed WM, Francis SS, Ajrouche R, Erdmann F, Orsi L, Spector LG (2016). Caesarean delivery and risk of childhood leukaemia: a pooled analysis from the Childhood Leukemia International Consortium (CLIC). Lancet Haematol.

[2] Linet MS, Brown LM, Mbulaiteye SM, Check D, Ostroumova E, Landgren A, Devesa SS (2016). International long-term trends and recent patterns in the incidence of leukemias and lymphomas among children and adolescents ages 0–19 years. Int J Cancer.

[3] Miller KD, Fidler-Benaoudia M, Keegan TH, Hipp HS, Jemal A, Siegel RL (2020). Cancer statistics for adolescents and young adults, 2020. CA Cancer J Clin.

[4] Inaba H, Mullighan CG (2020). Pediatric acute lymphoblastic leukemia. Haematologica.

[5] Ishii E. Hematological disorders in children: Pathogenesis and treatment. Springer; 2017.

[6] Wang J, Shen N, Zhang X, Shen M, Xie A, Howell D, Yuan C (2017). Care burden and its predictive factors in parents of newly diagnosed children with acute lymphoblastic leukemia in academic hospitals in China. Support Care Cancer.

[7] Muskat B, Jones H, Lucchetta S, Shama W, Zupanec S, Greenblatt A (2017). The experiences of parents of Pediatric patients with Acute Lymphoblastic Leukemia, 2 months after completion of treatment. J Pediatr Oncol Nurs.

[8] Chen J, Liu Y, Cai QQ, Liu YM, Wang T, Zhang K, Wang JF, Chen WQ, Huang H (2015). Type D personality parents of children with leukemia tend to experience anxiety: the mediating effects of social support and coping style. Med (Baltim).

[9] Wakefield CE, McLoone JK, Butow P, Lenthen K, Cohn RJ (2011). Parental adjustment to the completion of their child’s cancer treatment. Pediatr Blood Cancer.

[10] Lazarus RS, Folkman S (1987). Transactional theory and research on emotions and coping. Eur J Pers.

[11] Lazarus RS (1993). From psychological stress to the emotions: a history of changing outlooks. Ann Rev Psychol.

[12] Ellis KR, Janevic MR, Kershaw T, Caldwell CH, Janz NK, Northouse L (2017). Meaning-based coping, chronic conditions and quality of life in advanced cancer & caregiving. Psychooncology.

[13] Poręba-Chabros A, Kolańska-Stronka M, Mamcarz P, Mamcarz I (2022). Cognitive appraisal of the disease and stress level in lung cancer patients. The mediating role of coping styles. Support Care Cancer.

[14] Ravindran O, Shankar A, Murthy T (2019). A comparative study on perceived stress, coping, quality of life, and hopelessness between cancer patients and survivors. Indian J Palliat Care.

[15] Schneider TR (2008). Evaluations of stressful transactions: what’s in an appraisal?. Stress Health.

[16] Geerdink LM, Verhaak CM, Kapusta L (2021). Illness cognition and parenting stress in parents of children with Ebstein’s anomaly. J Psychosom Res.

[17] Hudson JL, Bundy C, Coventry PA, Dickens C (2014). Exploring the relationship between cognitive illness representations and poor emotional health and their combined association with diabetes self-care. A systematic review with meta-analysis. J Psychosom Res.

[18] Cuviello A, Raisanen JC, Donohue PK, Wiener L, Boss RD (2021). Initiating Palliative Care referrals in Pediatric Oncology. J Pain Symptom Manage.

[19] Van Schoors M, De Paepe AL, Lemiere J, Morez A, Norga K, Lambrecht K, Goubert L, Verhofstadt LL (2019). Family Adjustment when facing Pediatric Cancer: the role of parental psychological flexibility, Dyadic Coping, and Network Support. Front Psychol.

[20] Abera M, Robbins JM, Tesfaye M (2015). Parents’ perception of child and adolescent mental health problems and their choice of treatment option in southwest Ethiopia. Child Adolesc Psychiatry Ment Health.

[21] Levine DR, Mandrell BN, Sykes A, Pritchard M, Gibson D, Symons HJ, Wendler D, Baker JN (2017). Patients’ and parents’ needs, attitudes, and perceptions about early Palliative Care Integration in Pediatric Oncology. JAMA Oncol.

[22] Sisk BA, Kang TI, Mack JW (2020). The evolution of regret: decision-making for parents of children with cancer. Support Care Cancer.

[23] Verhoof EJ, Maurice-Stam H, Heymans HS, Evers AW, Grootenhuis MA (2014). Psychosocial well-being in young adults with chronic illness since childhood: the role of illness cognitions. Child Adolesc Psychiatry Ment Health.

[24] Han J, Liu JE, Qiu H, Nie ZH, Su YL (2018). Illness cognitions and the associated socio-demographic and clinical factors in Chinese women with breast cancer. Eur J Oncol Nurs.

[25] Coutu MF, Durand MJ, Baril R, Labrecque ME, Ngomo S, Côté D, Rouleau A (2008). A review of assessment tools of illness representations: are these adapted for a work disability prevention context?. J Occup Rehabil.

[26] Sint Nicolaas SM, Schepers SA, van den Bergh EMM, Evers AWM, Hoogerbrugge PM, Grootenhuis MA, Verhaak CM (2016). Illness cognitions and family adjustment: psychometric properties of the illness cognition questionnaire for parents of a child with cancer. Support Care Cancer.

[27] Zhang L, Zhou Y, Yang F, Han J (2023). Reliability and validity of Chinese Version of Illness Cognition Questionnaire-Parents in parents of children with Cancer. J Nurses Train.

[28] Willard VW, Hostetter SA, Hutchinson KC, Bonner MJ, Hardy KK. Benefit finding in maternal caregivers of Pediatric Cancer survivors: a mixed methods Approach. J Pediatr Oncol Nurs. 2016;33(5):353–60. 10.1177/1043454215620119. 10.1177/1043454215620119 26811326

[29] Katzman BI, John R (2018). Adolescent Cancer survivors: a Literature Review of Psychological effects following remission. Clin J Oncol Nurs.

[30] López J, Ortiz G, Noriega C. Posttraumatic growth in parents of children and adolescents with cancer. Sist Sanit Navar 42(3): 325–37. 10.23938/ASSN.0717 (Spanish). 10.23938/ASSN.0717 31859265

[31] Turner-Sack AM, Menna R, Setchell SR, Maan C, Cataudella D (2016). Psychological Functioning, post-traumatic growth, and coping in parents and siblings of adolescent Cancer survivors. Oncol Nurs Forum.

[32] Liu Q, Petrini MA, Luo D, Yang BX, Yang J, Haase JE (2021). Parents’ experiences of having a young child with Acute Lymphoblastic Leukemia in China. J Pediatr Oncol Nurs.

[33] Wu HF, Bi XY, Li J, Wang JT (2020). Analysis on care ability and its influencing factors for parents of children with leukemia. Nurs J Chin People’s Liberation Army.

[34] Srivastava S, Menon V, Kayal S, Hari M, Dubashi B (2020). Level of anxiety and depression and its clinical and sociodemographic determinants among the parents of children with Cancer Undergoing Chemotherapy. J Neurosci Rural Pract.

[35] Santacroce SJ, Kneipp SM (2020). Influence of pediatric cancer-related financial burden on parent distress and other stress-related symptoms. Pediatr Blood Cancer.

[36] Nam GE, Warner EL, Morreall DK, Kirchhoff AC, Kinney AY, Fluchel M (2016). Understanding psychological distress among pediatric cancer caregivers. Support Care Cancer.

[37] Tanner L, Keppner K, Lesmeister D, Lyons K, Rock K, Sparrow J (2020). Cancer Rehabilitation in the Pediatric and Adolescent/Young Adult Population. Semin Oncol Nurs.

[38] Woodson KD, Thakkar S, Burbage M, Kichler J, Nabors L (2015). Children with chronic illnesses: factors influencing family hardiness. Issues Compr Pediatr Nurs.

[39] Al-Gamal E, Long T (2010). Anticipatory grieving among parents living with a child with cancer. J Adv Nurs.

[40] Schwartz-Attias I, Krulik T, Amit Aharon A, Ronen T (2023). Perceptions of children with cancer and their parents regarding illness: a qualitative study. J Pediatr Nur.

[41] Seth R, Singh A (2015). Leukemias in Children. Indian J Pediatr.

[42] Malard F, Mohty M (2020). Acute lymphoblastic leukaemia. Lancet.

[43] Nakayama N, Mori N, Ishimaru S, Ohyama W, Yuza Y, Kaneko T, Kanda E, Matsushima E (2017). Factors associated with posttraumatic growth among parents of children with cancer. Psycho-oncology.

[44] Wikman A, Mattsson E, von Essen L, Hovén E (2018). Prevalence and predictors of symptoms of anxiety and depression, and comorbid symptoms of distress in parents of childhood cancer survivors and bereaved parents five years after end of treatment or a child’s death. Acta Oncol.

[45] Shattnawi KK, Okour H, Alnatour A, Al-Sheyab N, Mrayan L, Joseph RA (2021). Caring for a child with Cancer: experiences of Jordanian mothers. Clin Nurs Res.

[46] Woodgate RL, Tailor K, Yanofsky R, Vanan MI (2016). Childhood brain cancer and its psychosocial impact on survivors and their parents: a qualitative thematic synthesis. Eur J Oncol Nurs.

[47] LiuYN, Li HY, Huang HY (2021). Status of anticipatory grief among main caregivers of childhood hematopathy and its influencing factors. Chin Nurs Res.

